# Case Report: Persistent Pulmonary Hypertension of the Newborn and Narrowing of the Ductus Arteriosus After Topical Use of Non-Steroidal Anti-Inflammatory During Pregnancy

**DOI:** 10.3389/fphar.2021.756056

**Published:** 2021-11-25

**Authors:** Kévin Le Duc, Sixtine Gilliot, Jean Benoit Baudelet, Sébastien Mur, Mohamed Riadh Boukhris, Olivia Domanski, Pascal Odou, Laurent Storme

**Affiliations:** ^1^ Department of Neonatology, Jeanne de Flandre Hospital, University Hospital of Lille, Lille, France; ^2^ ULR2694 Metrics–Perinatal Environment and Health, University of Lille, Lille, France; ^3^ ULR 7365–GRITA–Groupe de Recherche sur Les Formes Injectables et Les Technologies Associées, Université de Lille, CHU Lille, Lille, France; ^4^ Institut de Pharmacie, CHU Lille, Lille, France; ^5^ Department of Pediatric Cardiology, Institut Coeur Poumon, University Hospital of Lille, Lille, France

**Keywords:** PPHN (persistent pulmonary hypertension of the newborn), NSAID (non-steroidal anti-inflammatory drug), ductus arteriosus, NICU (neonatal intensive care unit), pregnancy, neonate

## Abstract

**Background:** The use of non-steroidal anti-inflammatory drugs (NSAIDs) during the third trimester of pregnancy can cause premature constriction of the ductus arteriosus. This report describes a case of *in utero* narrowing of the ductus arteriosus (DA) diagnosed postnatally in a baby with Persistent Pulmonary Hypertension of the Newborn (PPHN), after maternal use of Diclofenac-Epolamine 140 mg patch during the second and third trimester.

**Case Presentation:** A fetal ultrasounds revealed an enlarged hypertrophic right ventricle at 32 weeks of gestation. Detailed questioning of the mother highlighted that topical Diclofenac (FLECTOR^®^) had been used at 26 and at 31 weeks of gestation. An echocardiography performed 8 h postnatally showed supra-systemic pulmonary hypertension, a restrictive ductus arteriosus and a dilated right ventricle. The newborn was treated by inhaled nitric oxide and oral Sildenafil and was discharged from hospital on day 24. He had a complete normalization of his pulmonary vascular resistance on day 48.

**Conclusion:** This case illustrates the potential fetal and neonatal complications associated with maternal topical Diclofenac medication during pregnancy resulting in antenatal closure of the DA.

## Introduction

During pregnancy, the right ventricular output is mostly directed from the pulmonary artery to the aorta, which contributes to systemic circulation (Rasanen et al., 1996). Patency of the ductus arteriosus is maintained during gestation by an elevated concentration of prostaglandin (PGE_2_) and a low fetal blood partial pressure of O_2_ (Clyman, 2006).

The use of non-steroidal anti-inflammatory drugs (NSAIDs) during the third trimester of pregnancy can cause premature constriction of the ductus arteriosus by inhibiting cyclo-oxygenase 2 (COX-2) (Van Marter et al., 1996; Gewillig et al., 2009; Storme et al., 2013). Diclofenac, a NSAID, was found to show the greatest potency for inhibition of phorbol ester-induced PGE_2_ production (reflecting inflammation induced COX-2 activity) compared with a similar range of NSAIDs in human fibroblasts *in vitro*. It has also been classified as one of the most potent inducers of ductus arteriosus constriction in rats (Momma et al., 1984; Cordero et al., 2001). Diclofenac is a low molecular weight molecule of 318.15 Da which crosses trophoblastic membranes easily, with a mean maternal/fetal drug ratio that is inferior to one, indicating the drug may accumulate in fetal tissue over time (Siu et al., 2000). The placental transport of NSAIDs involves specific transports including monocarboxylate transporter 4 -a proton dependent transporter, which transports L-lactic acid as a substrate (Emoto et al., 2002). This case report highlights the importance of informing pregnant women about the risk of self-medication and topical NSAID use during pregnancy.

## Case Description

**FIGURE 1:**
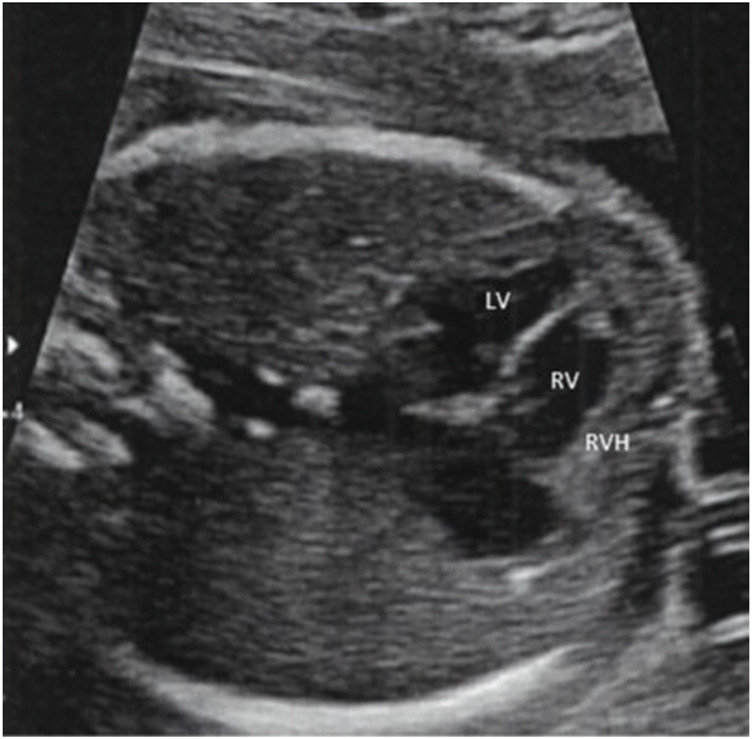
Apical 4 chamber during fetal echocardiography at 32 weeks of gestation. Cardiac ventricular asymmetry and right ventricle cardiac hypertrophy. LV: Left Ventricle; RV: Right Ventricle; RVH: Right Ventricle Hypertrophy.

We report the case of a newborn infant with persistent pulmonary hypertension of the newborn (PPHN) resulting from antenatal narrowing of the ductus arteriosus related to maternal application of topical Diclofenac during the second trimester of pregnancy. The infant was born to a 35-year-old mother both the course of the pregnancy–the mother’s second–and antenatal blood tests were unremarkable. As recommended by the French National technical Committee on Prenatal Ultrasound Screening, three antenatal echography were performed during the first, second were described as normal. The third revealed cardiac ventricular asymmetry and a right ventricle cardiac hypertrophy (Figure 1).

**FIGURE 2:**
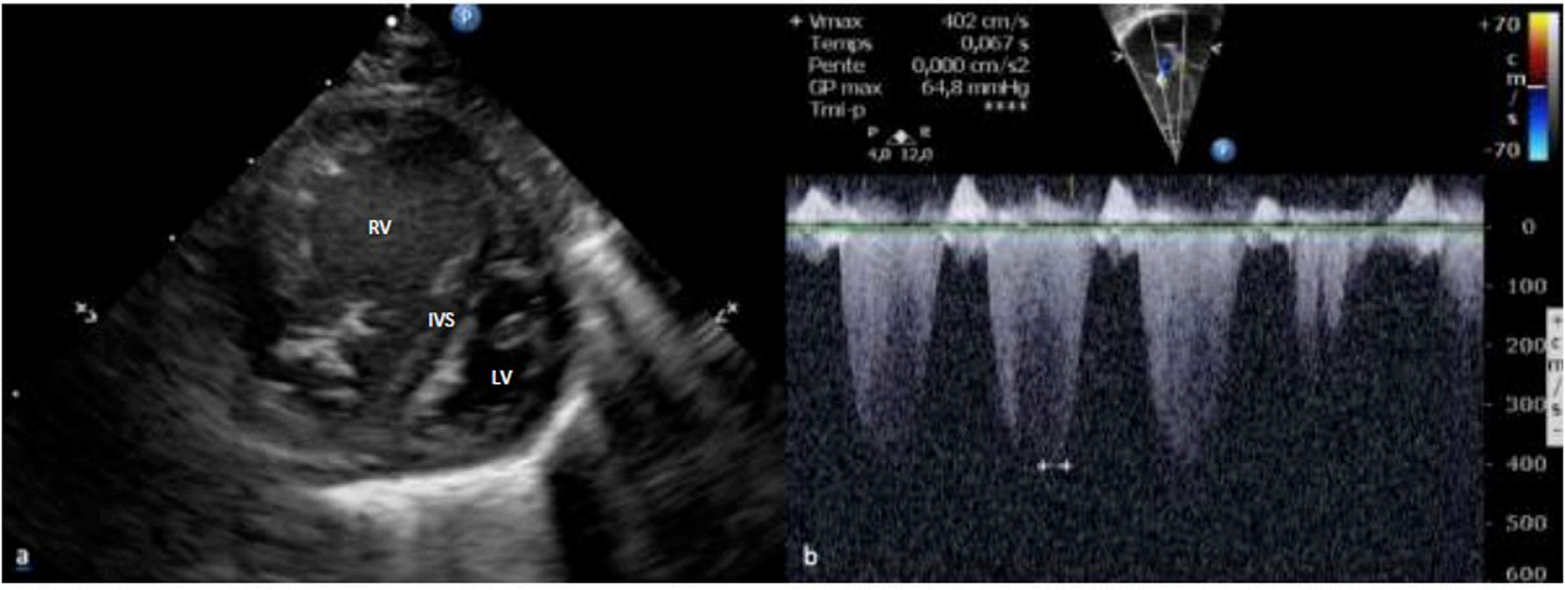
Supra-systemic pulmonary hypertension was assessed on echocardiography. Parasternal short axis ventricles **(A)**: Bowing of the interventricular septum into the left ventricle. Apical 4 chamber **(B)**: Tricuspid regurgitation blood flow velocities higher than 4 m.s^−1^. RV: Right Ventricle; LV: Left Ventricle; IVS: Interventricular Septum.

A male infant weighing 3,470 g (50th-90th percentile) was delivered at 39 weeks of gestation by a planned cesarean section for breech presentation. Apgar scores were 10 at both 1 and 5 min. A pediatrician examined the baby 8 h after birth for moderate dyspnea. Cardiac auscultation revealed a systolic ejection murmur loudest over the pulmonary valve Pre-ductal oxygen saturation was 88% in room air, which increased to 98% with high flow nasal cannula at 2 L/Kg/min and O_2_ supplementation (from 30 to 100% FiO_2_). The newborn was admitted to the Neonatal Intensive Care Unit where an echocardiography showed supra-systemic pulmonary hypertension with tricuspid regurgitation blood flow velocities of 60 mmHg (Figure 2), whereas systolic blood pressure was 54 mmHg. An accelerated right-to-left shunting was recorded across a restrictive DA. No cardiac malformation was found.

Our hypotheses were therefore:

- a premature closure of the ductus arteriosus during pregnancy,
- a premature closure of the foramen ovale,
- or an alveolar capillary dysplasia.

A CT scan with contrast was performed 5 days after birth, showing an aneurysm of the inter-atrial septum without shunting, a dilated right ventricle and a markedly dilated main pulmonary artery.

**FIGURE 3:**
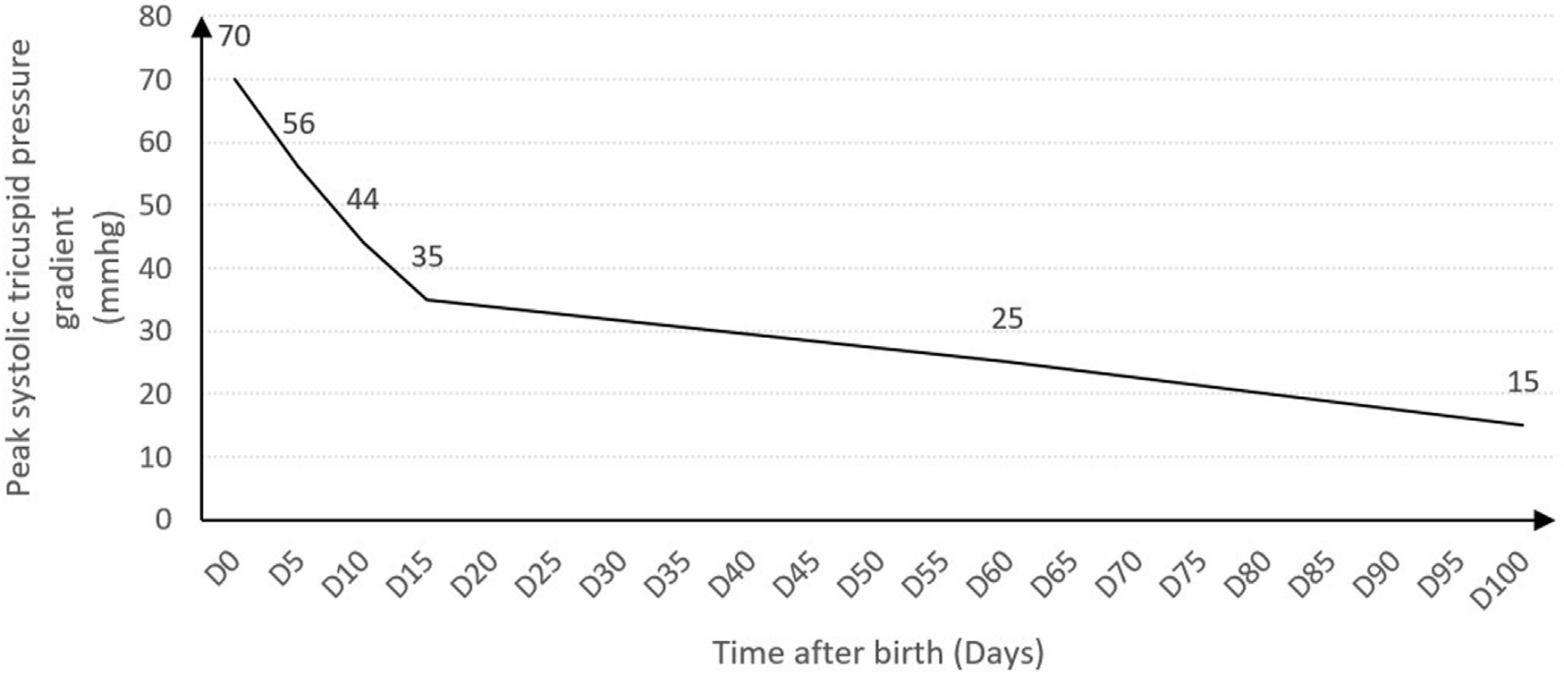
Change of pulmonary artery systolic pressures (mmHg) estimated by measuring peak systolic tricuspid regurgitation velocity. PAPs normalized 2 months after birth. D, Day.

The newborn was managed by inhaled NO (20 ppm), alprostadil infusion (0.02 µg^−1^ kg.min^−1^) and high-flow oxygen supplementation through nasal cannulas for 18 days. Oral Sildenafil was started on day 3 due to the persistence of pulmonary hypertension. Symptoms improved on day 17, the ductus arteriosus was closed at day 20 and the infant was discharged from hospital on day 24 with normalization of pulmonary pressures (Figure 3). Sildenafil was stopped on day 157. At the age of 1 year, the infant presented no symptoms, a normal neuro-developmental outcome and a normal echocardiography.

Rigorous and repeated anamnestic determined that the mother applied Flector^®^ (Diclofenac-Epolamine 140 mg patch, GENEVRIER SA Laboratory) on her lumbar area for back pain at 26 and 31 weeks of gestational age. In order to further reduce the pain, a hot water bottle was also applied to the area at the same time as the patch.

## Discussion

This case reveals the risk of a sustained narrowing of the ductus arteriosus resulting in prolonged persistent pulmonary hypertension in the postnatal period after maternal use of topical diclofenac during the second trimester.

Besides NSAIDs, other causes of fetal ductal constriction and PPHN have previously been reported. Acetaminophen may increase the risk of prenatal ductus arteriosus constriction, through inhibition of prostaglandin G2 synthesis. Growing body of evidence suggests a role of maternal consumption of polyphenols-rich food (green tea, orange juice, coco bean, spring vegetables), which interfere with prostaglandin metabolism, in prenatal ductus arteriosus narrowing (Zielinsky and Busato, 2013). After rigorous questioning of the mother, no paracetamol treatment or excessive polyphenol-rich food intake were recorded.

Antenatal narrowing of the DA results in increased pulmonary artery pressure, which in turn mediates a remodeling of the vascular wall through sustained elevation of vascular stretch stress, leading to structural pulmonary hypertension (Storme et al., 2002; Larrue et al., 2005; Shima et al., 2011).

Previous studies clearly showed that DA sensitivity to constricting factors increases during the last trimester of pregnancy (Van den Veyver and Moise, 1993). The risk of indomethacin-induced fetal ductal constriction increases with advancing gestational age (Luchese et al., 2003). Second trimester fetal adverse events have been reported after prolonged NSAID exposure of at least 7 days while our case reports the occurrence of DA narrowing after two topical applications of a daily NSAID patch (48 h of total application) (Dathe et al., 2019).

Only one previous case of antenatal DA closure has been reported after maternal topical use of diclofenac during the third trimester of pregnancy. In that case, diclofenac has been applied at 35 weeks gestational age and the constriction of the ductus arteriosus was rapidly reversible at birth (Torloni et al., 2006). In this latter case report, the formulation of diclofenac increminated in DA narrowing was a gel formulation of diclofenac diethlyamine (Cataflam Rmugel 11.6 mg/g, Novartis). It is of current knowledge that topical NSAIDs, including diclofenac diethylamine, are associated with systemic effects (Evans et al., 1995). The patch delivery in diclofenac-epolamine (DI-EP) was developed more recently to prevent the acute release of NSAIDS to the bloodstream and control the amount of active substance delivered through the skin, in contrast to that from application of bioequivalent gels or ointments of diclofenac (Goh and Lane, 2014). The pharmacokinetics parameters of diclofenac patches are expected to lengthen the time of product release in the central comportment, to reach a maximal plasmatic concentration of about 20 ng/ml (Rainsford et al., 2008). Previous studies on transplacental pharmacokinetics of diclofenac reported that the fetal peak plasma concentration was estimated to be one-tenth of the maternal value (Shintaku et al., 2012). Concerning pharmacodynamics, an animal predictive model performed on rats revealed that the concentration–response (response being DA narrowing) relationship in rat fetus was characterized by an EC50 of 1.4 ng/ml, reinforcing the plausibility of the link between diclofenac patch application and DA narrowing in our case report (Shintaku et al., 2012). Furthermore, it is likely that the use of hot water bottle for back pain may have promoted skin absorbance of the drug (Park et al., 2008; Panda et al., 2019).

There are no reports on the occurrence of systemic side effects related to a single topical NSAIDs application, when applied in conformity with recommendations of us, in current literature. The risk of occurrence is often related to misuse; in our case, the application of heat to the same area as the patch led to an increase of the drug’s absorption and the modification of the delivery profile of diclofenac.

Regarding these elements, the implication of the DI-EP patch (Flector^®^) in the occurrence of pulmonary hypertension in newborn infants cannot be excluded. Furthermore, it is likely that the use of a hot water bottle for back pain may have promoted skin absorbance of the drug (Park et al., 2008; Panda et al., 2019). In light of this evidence, the role of the DI-EP patch (Flector^®^) on pulmonary hypertension in newborns in must be highly considered.

Cases of antenatal closure of the DA after oral use of diclofenac during the third trimester of pregnancy have more commonly been reported (Auer et al., 2004). According to the summary of product characteristics, the use of FLECTOR^®^ patch must be avoided during the five first months of pregnancy and is strictly contraindicated during the 3rd trimester of pregnancy. Many other patches containing diclofenac as the main active substance are available on the market (Voltarenplast^®^, Antacalm^®^, Flectoreffigel^®^). They differ in the salt of diclofenac they contain and must be responsible for an equivalent risk of DA narrowing. Patches containing diclofenac salts are over-the-counter medications in many countries and their use does not require any medical prescription, enhancing the risk of misuse in pregnant women.

Our findings suggest that maternal educational programs should include information pertaining to topical treatments in order to prevent potentially harmful self-medication in pregnant women.

## Data Availability

The raw data supporting the conclusions of this article will be made available by the authors, without undue reservation.
